# Pregnancy AI: Development and Internal Validation of an Artificial Intelligence Tool to Predict Live Births in ICSI and IVF Cycles Using Clinical Features and Embryo Images

**DOI:** 10.3390/medicina62020364

**Published:** 2026-02-12

**Authors:** Jaume Minano Masip, Penelope Borduas, Isaac-Jacques Kadoch, Simon Phillips, Doina Precup, Daniel Dufort

**Affiliations:** 1 Département d’Obstétrique et de Gynécologie, Faculté de Médecine, Université de Montréal, Montréal, QC H3T 1J4, Canada; 2 Department of Obstetrics and Gynecology, Centre Hospitalier de l’Université de Montréal, Montréal, QC H2X 3E4, Canada; 3 Division of Experimental Medicine, McGill University, Montréal, QC H3A 2T5, Canada; daniel.dufort@mcgill.ca; 4 Clinique Ovo, Montréal, QC H4P 2S4, Canada; 5 Department of Computer Science, McGill University, Montréal, QC H3A 2T5, Canada; 6 Child Health and Human Development Program, Research Institute of the McGill University Health Centre, Montréal, QC H4A 3J1, Canada; 7 Department of Obstetrics and Gynecology, McGill University, Montréal, QC H3A 2T5, Canada

**Keywords:** artificial intelligence, predictive model, time to pregnancy, embryo time-lapse imaging, in vitro fertilization

## Abstract

*Background and Objectives:* This study aimed at developing an AI-based predictive model for live birth based on a combination of a support vector machine (SVM) using clinical and embryological features, together with a convolutional neural network (CNN) using embryo time-lapse videos. *Materials and Methods:* This was a retrospective cohort analysis. Two hundred fifty-nine infertile couples treated between January 2012 and December 2019, with a total of 2330 embryos, were included in this study, and clinical data and images from 355 transferred embryos were used to build a predictive model. The main outcome was accuracy of live birth prediction. The secondary outcomes included accuracy in the prediction of biochemical pregnancy, clinical pregnancy and transferrable embryos. *Results*: The model was able to predict the transferrable embryo (i.e., embryos suitable for transfer or cryopreservation) with an accuracy of 0.98 in an internal set. The accuracy for predicting live birth, clinical pregnancy, and biochemical pregnancy exclusively using clinical data as input for an SVM model was 0.67, 0.68, and 0.67, respectively. With six frames from time-lapse embryo development, the CNN produced an accuracy of 0.57, 0.67, and 0.72. The predictive model performed best when combining input from clinical data and images from multiple embryo developmental frames, obtaining 0.71, 0.73, and 0.77 for predicting live birth, clinical pregnancy, and biochemical pregnancy. *Conclusions*: This study highlights the potential of combining clinical data and embryo development images to enhance predictive models in reproductive medicine.

## 1. Introduction

In recent years, there has been a significant increase in the demand for assisted reproductive technology (ART) worldwide, with more couples turning to in vitro fertilization (IVF) [1]. The timing for conception is critical in fertility. Age correlates with declining reproductive capacity due to diminished ovarian reserve and poor oocyte quality, among other factors. Accelerating the time to conception with fewer IVF cycles and/or embryo transfers allows individuals to maximize their fertility potential before facing these exacerbated declines while mitigating the financial burden associated with fertility treatments [2]. Additionally, a shorter time to pregnancy can alleviate the emotional strain associated with ARTs, supporting the well-being of couples facing infertility.

Embryo scoring is a routine clinical task performed by embryologists to evaluate the morphological quality of embryos [3]. The accuracy of this process is critical because embryos will be transferred, frozen, or discarded depending on their score. Manual embryo scoring is time-consuming and relies on highly trained embryologists, which can incur subjective variability [4]. Despite the development of several scoring criteria, identifying the embryos that will most likely lead to pregnancy remains a challenge in assisted reproduction laboratories. Consequently, there has been increasing interest to utilize new technologies, such as artificial intelligence (AI), to improve embryo selection and maximize the chances of pregnancy [5].

Artificial intelligence has emerged as a transformative tool in various fields of medicine, offering unprecedented opportunities for personalized healthcare and precision medicine. AI can match and outperform human expertise in assessing medical imaging [6,7]. In reproductive medicine, AI has a wide range of applications including semen analysis [8], the management of ovarian stimulation [9], oocyte and embryo evaluation [10], automated blastocyst annotation [11], prediction of implantation [12], and non-invasive screening for ploidy [13].

Predicting pregnancy outcomes have focused on integrating embryo images, either static or timelapse, with detailed clinical features. The most common algorithms include Convolutional Neural Networks (CNNs), Multi-Layer Perceptrons (MLPs), Attention Branch Networks (ABNs) and fusion models that integrate both embryo images and clinical features, achieving the highest predictive performance. However, most models report clinical pregnancy as their endpoint, which does not always translate to live birth. Predicting live birth is more challenging due to additional confounding factors post-implantation. Another limitation is that several models, especially CNNs, often function as “black boxes,” making it difficult to understand the rationale behind specific predictions, though attention mechanisms and visualization tools are improving this aspect. Finally, the exclusion of “poor quality” embryos from training datasets may reduce the robustness of models and their ability to generalize to all embryo types.

In this retrospective cohort study, we integrated 35 clinical features, including IVF cycle characteristics, with time-lapse embryo development images into a machine learning-based predictive model designed for clinicians and embryologist to help optimize embryo selection in both IVF and ICSI cycles in all patients and enhance live birth outcomes within a shorter timeframe.

## 2. Materials and Methods

This retrospective cohort study was conducted at a university-affiliated fertility clinic in Montreal from January 2016 to December 2019. The study was approved by the OVO R&D Scientific Review Committee (#080920). All participants provided written informed consent.

### 2.1. Study Design

**Figure 1:**
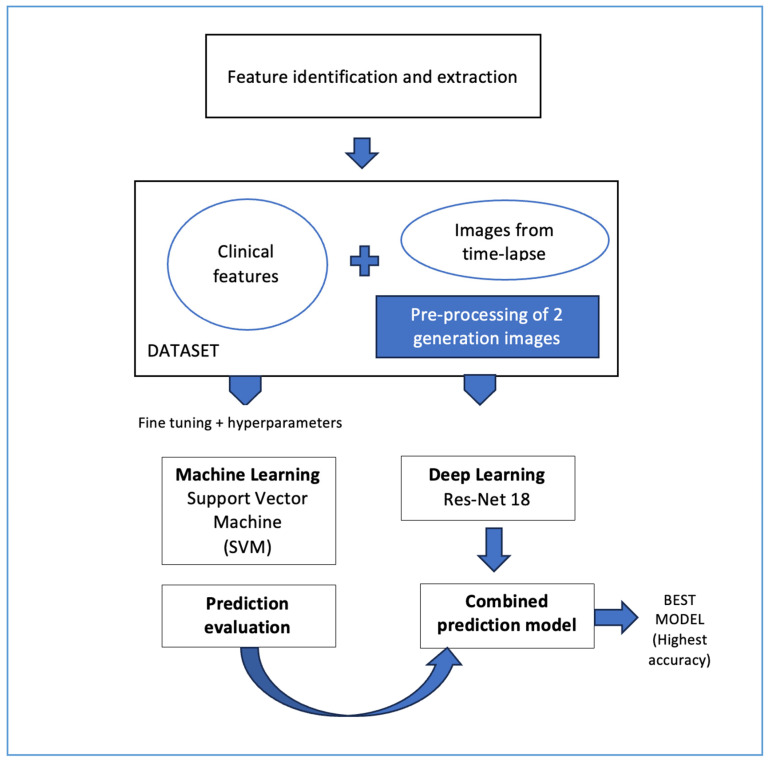
Model development and optimization.

The study included 259 women undergoing IVF who collectively produced 2330 embryos of which 355 developed to transferred blastocysts. Inclusion criteria included women who underwent in vitro fertilization (IVF) or intracytoplasmic sperm injection (ICSI) treatment between January 2012 and December 2019 in a single clinic. No restriction was applied regarding the type of ovarian stimulation protocol, including gonadotropin-releasing hormone (GnRH), antagonist protocols, and short protocol. Embryo culture was performed using the Geri^®^ time-lapse incubator system, Genea Biomedx, Sydney, Australia. The sperm source was either from a male partner or a donor, and was obtained either via ejaculation or testicular biopsy. Female patients were younger than 44 years at the time of oocyte retrieval. Finally, the patients had to have at least one blastocyst available for transfer. Exclusion criteria included patients who declined the use of time-lapse imaging technology for embryo culture. PGT-A was not performed in any of the cycles. Patients were categorized into two groups based on the insemination method: standard IVF or intracytoplasmic sperm injection (ICSI). To develop a comprehensive AI model that can select embryos with the highest potential for achieving a live birth, we integrated a support vector machine (SVM) learning model for clinical data with a convolutional neural network (CNN), applying deep learning to embryo images (Figure 1).

### 2.2. Ovarian Stimulation Characteristics

Ovarian stimulation protocols were carried out with the gonadotropin-releasing hormone (GnRH) antagonist protocol, the short-acting or long-acting agonist protocol, or the SMART (Stimulation with Minimal Adverse Effects, Retrieval, and Transfer) protocol. The first three protocols employed various gonadotropins including follitropin alfa (Puregon^®^, Puregon^®^, Merck Canada Inc., Kirkland, QC, Canada), follitropin beta (Gonal-F^®^, Toronto, ON, Canada), and highly purified human menopausal gonadotropin (HP-hMG; Menopur^®^, Ferring Toronto, ON, Canada).

In GnRH antagonist cycles, gonadotropin administration commenced on day 3 of the menstrual cycle. Subcutaneous injections of 0.25 mg cetrorelix (Cetrotide^®^, EMD Serono Canada, Mississauga, ON, Canada) or ganirelix acetate (Orgalutran^®^, Organon Canada, Kirkland, QC, Canada) were initiated when the leading follicle measured > 12 mm or estradiol levels exceeded 2000 pmol/L. This regimen continued until the ovulation trigger with 5000 IU human chorionic gonadotropin (hCG^®^, EMD Serono Canada, Mississauga, ON, Canada) or triptorelin (Decapeptyl 0.1 mg/mL, Ferring, Toronto, ON, Canada). in patients with a risk of ovarian hyperstimulation syndrome. In all cases, the daily doses of gonadotropins ranged between 150 and 450 IU.

The short-acting GnRH agonist protocol involved initiating subcutaneous buserelin acetate (Suprefact^®^, Ferring, Toronto, ON, Canada) at 0.05 mg twice a day, from day 2 of the cycle on the same day of gonadotropins until the ovulation triggers with subcutaneous administration of 5000 IU hCG (EMD Serono Canada, Mississauga, ON, Canada).

In contrast, the long-acting GnRH agonist protocol involved initiating subcutaneous administration of 0.05 mg buserelin acetate (Suprefact^®^, Ferring Toronto, ON, Canada) twice a day during the mid-luteal phase of the preceding natural menstrual cycle. After at least 14 days of the GnRH agonist, the dose was decreased to 0.05 mg daily when the gonadotropin was introduced.

The SMART protocol was initiated on day 20 of the preceding cycle with 4 mg of oral 17β-Oestradiol (Estrace) daily. On day 2 of the subsequent menstruation, 5 mg of letrozole (Femara^®^, Novartis Pharma Canada, Dorval, QC, Canada) was added daily. Gonadotropins were administered from day 4 to the trigger day, using 300–450 IU HP-hMG (Menopur^®^, Ferring, Toronto, ON, Canada). GnRH antagonists were initiated when the dominant follicle reached a ≥14 mm diameter. Ovulation was triggered using subcutaneous injections of hCG (Ovidrel^®^, EMD Serono Canada, Mississauga, ON, Canada) or Suprefact^®^ (Ferring, Toronto, ON, Canada).

In all four protocols, the response to stimulation was monitored through serum estradiol (E2) levels and vaginal ultrasound. Ovulation was triggered when there were at least three antral follicles with a diameter ≥ 18 mm present. Oocyte retrieval was scheduled 36 ± 2 h thereafter.

### 2.3. In Vitro Fertilization Characteristics

Depending on the clinical evaluation, oocytes were inseminated with the partner’s or donated sperm using standard IVF or ICSI. Partner sperm was obtained through ejaculation or testicular aspiration. Semen quality was assessed following the 5th World Health Organization guideline [14], evaluating parameters such as semen volume, concentration, and motility.

### 2.4. Time-Lapse Embryo Culture Images

Following IVF or ICSI, oocytes were individually cultured with pre-equilibrated continuous media within a time-lapse incubator system (Geri^®^, Genea Biomedx, Sydney, Australia) at 37 °C, with 5% O_2_ and 6% CO_2_. Images of the embryos were captured every five minutes across 11 focal planes. However, only the best-focused plane (automatically detected by the incubator algorithm) was used for the study. Six frames representing key embryo development stages (0 h, 18 h, 48 h, 72 h, 120, final) were selected based on the routine IVF laboratory practices for evaluating embryo development.

### 2.5. Reproductive Outcome Measures

Blastocysts were evaluated by embryologists on day 5 or 6 using the Gardner classification system. Viable blastocysts with an expansion grade between 2 and 4, high-quality inner cell mass and grade A/B trophectoderm were either transferred fresh or vitrified. Blastocysts not meeting these criteria were discarded. Reproductive outcome measures included live birth, clinical pregnancy, and biochemical pregnancy following a single embryo transfer (SET). Biochemical pregnancy was defined as two consecutive serum β-hCG measurements ≥ 5 IU/L, obtained 10 to 14 days after embryo transfer SVM. Clinical pregnancy was confirmed by detecting a fetal heartbeat during the 7-week ultrasound scan.

### 2.6. Building the Dataset

#### 2.6.1. Clinical Feature Pre-Processing

To develop the predictive models, we used a total of 35 clinical variables encompassing demographic, hormonal, lifestyle, cycle, and gamete parameters from both partners. These features were selected based on clinical relevance, data availability, and prior evidence of association with fertility outcomes (Supplementary Table S1).

Categorical features were converted into a series of binary variables using one-hot encoding. Continuous variables were normalized using z-score normalization (i.e., subtracting the mean and dividing by the standard deviation). For variables with missing values, we applied mean imputation when the proportion of missing data was <10%.

Inclusion threshold: Variables with >30% missing data were excluded from the model to preserve data quality (none of the final variables included exceeded this threshold).

#### 2.6.2. Image Pre-Processing and Normalization

**Figure 2:**
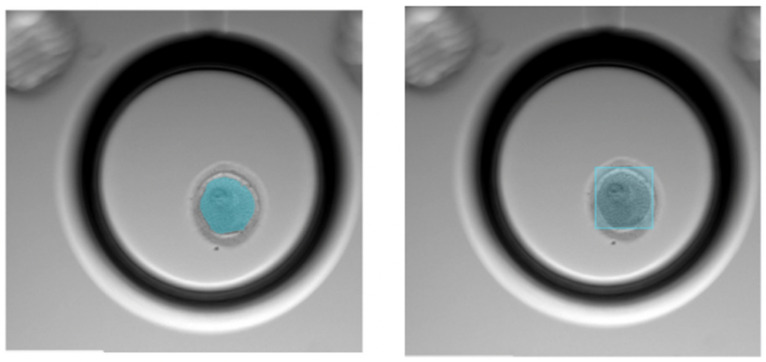
Embryo image annotation. (**Left**: Pre-Boxing, **right**: Post-Boxing). The blue overlay represents the segmented embryo (region of interest), and the blue frame indicates the automated bounding box used to crop and standardize the image for analysis.

The “best-focused plane” was selected using the Geri^®^ time-lapse system’s built-in automated algorithm, which identifies the sharpest image among 11 focal planes based on image clarity metrics such as contrast and edge sharpness. In addition to the clinical features, the six key embryo development time-lapse images were also used to train the AI model. Due to the time-lapse incubator’s evolving software, the images were stored in various formats. The first-generation incubator captured images at 1442 × 1442 pixels, with the embryo occupying a small portion of the image. In contrast, the second-generation system recorded zoomed-in videos of the embryo at 512 × 512 pixel resolution. To adapt to these differences, 512 × 512 pixel regions containing the embryo were automatically cropped from each 1442 × 1442 pixel image using a modified Fast R-CNN object detector [15]. Fast R-CNN detects and classifies salient objects in images using a candidate-proposing network and a predefined list of categories, respectively. For this study, we optimized the Fast R-CNN to specifically detect and crop the time-lapse images using 100 manually annotated images with embryos present (Figure 2). To avoid color biases and normalize the image brightness, the RGB color values of the 512 × 512 pixel images were normalized using ImageNet’s mean and standard deviation values.

#### 2.6.3. Data Partitioning

The patient dataset was randomly divided into three subsets: training (70%), validation (15%), and test (15%). The 15% validation set was used for hyperparameter tuning (internal validation), while external validation was not performed. To prevent any risk of data leakage, data partitioning was performed at the patient level. All embryos belonging to the same patient were assigned exclusively to a single subset (training, validation, or test). No patient contributed embryos to more than one subset. This ensured that embryo-level tasks were evaluated on patients entirely unseen during training, thereby preserving the independence of the test set and the validity of the performance estimates.

### 2.7. Building a Predictive Machine Learning Model

#### 2.7.1. Support Vector Machine (SVM)

The clinical-data model was implemented using a Support Vector Machine (SVM) classifier in Scikit-learn with probabilistic output. All features were standardized prior to training. Different kernels (linear, polynomial, and RBF) and regularization parameters were evaluated. Hyperparameters (C and γ) were optimized using stratified K-fold cross-validation with grid search. The final model used an RBF kernel and generated class probabilities using Platt scaling.

#### 2.7.2. Convolutional Neural Network (CNN)

The embryo image-based model was implemented using a convolutional neural network based on the Residual Network architecture (ResNet-18) in PyTorch 2.8.0 (pytorch.org; accessed on 20 August 2025). ResNet architectures use skip connections to facilitate gradient propagation and stabilize training in deep networks. We selected ResNet-18 as a trade-off between representational capacity and overfitting risk given the limited dataset size. The network was initialized with weights pretrained on the ImageNet dataset (image-net.org; accessed on 20 August 2025), which contains over one million natural images, and subsequently fine-tuned on embryo images using transfer learning.

The original classification head of ResNet-18 was replaced with a task-specific fully connected layer producing two outputs corresponding to the target classes (positive vs. negative pregnancy outcome). All images were resized to 256 × 256 pixels. During training, data augmentation was applied using random cropping to 224 × 224 pixels, while center cropping to 224 × 224 pixels was used during validation and testing to ensure deterministic evaluation.

The model was trained using the categorical cross-entropy loss function and optimized with the Adam optimizer. We empirically evaluated different learning rates, batch sizes, data augmentation strategies, and regularization techniques. Due to computational constraints, training was performed for a maximum of 100 epochs, with the final model selected based on validation performance.

Two image input configurations were evaluated: (1) a single time-point embryo image, and (2) a multichannel input formed by concatenating six key time-lapse frames along the channel dimension, resulting in an 18-channel input tensor. This design allows the network to implicitly learn spatiotemporal morphological patterns without explicit recurrent or temporal modeling. For each input sample, the network outputs a calibrated probability score for each target class.

Finally, predictions from the CNN embryo image-based model and the SVM clinical feature-based model were combined using a late fusion strategy by averaging the predicted class probabilities.

#### 2.7.3. Grad-CAM

Gradient-weighted Class Activation Mapping (Grad-CAM) was used as a qualitative interpretability tool to visualize the spatial regions of embryo images that contributed most to the CNN predictions. This approach allows visual assessment of whether the network focuses on biologically meaningful structures, such as the inner cell mass and trophectoderm, thereby improving model transparency and facilitating clinical interpretation of the learned representations. In this study, Grad-CAM was used exclusively for qualitative assessment of biological plausibility; quantitative region-based analyses were not performed and are left for future work.

### 2.8. Evaluation of Model Performance

The performance of our pregnancy AI model was evaluated based on the accuracy of correct classifications. The validation dataset was used to determine the best model hyperparameters while the test set was used to evaluate the model’s performance in four different scenarios (using only clinical data, a single or six key frames of embryo development, or a combination of the clinical data and the key frames). Our models were trained to predict embryo viability and a successful embryo transfer, considering various outcomes, including biochemical pregnancy, clinical pregnancy, and live birth.

### 2.9. Estimating the Reduction in Time to Pregnancy

As an indirect measure of how the time to pregnancy could be shortened, we considered the number of transfers necessary to achieve a live birth. We used the formula:

Cumulative Probability = (1− (1 − P)^N^) × 100

Which estimates the probability of achieving at least one live birth over *N* independent embryo transfers, assuming a constant success probability *P* per transfer. This is based on the binomial distribution, where the probability of at least one success in *N* Bernoulli trials is given by 1 − (1 − P)^N^. The cohort live birth rate was calculated as the proportion of single embryo transfers (SETs) that resulted in live births across the study dataset.

### 2.10. Statistical Analyses

Statistical differences in baseline characteristics were analyzed using *t*-tests for independent samples (Table 1, Table 2 and Table 3). In all cases, *p* < 0.05 was considered statistically significant.

### 2.11. Data Sharing

To promote transparency and reproducibility in science, the code will be made available upon request. To request code, please send an email to the first author.

## 3. Results

### 3.1. Baseline Characteristics and Reproductive Outcomes

Baseline female characteristics show a favorable prognostic profile (Table 1), with a mean age of 35 years, good ovarian reserve (anti-müllerian hormone > 2 ng/mL) and more than 15 antral follicles, along with a normal weight (body mass index < 25). Approximately one-third of patients reported alcohol consumption. The majority of patients (88.1%) underwent GnRH antagonist protocols. The short-acting GnRH agonist, long-acting GnRH agonist and SMART ovarian stimulation protocols were employed in 6.7%, 1.1%, or 4.1% of cases, respectively. Overall, the estradiol levels at the time of the ovulation trigger were maintained below 10,000 ng/mL while progesterone levels were below 4.7 ng/mL. There was a median of 15 oocytes retrieved per cycle, indicating optimal ovarian stimulation.

**Table 1:** Demographics and baseline reproductive characteristics of the female participants.

Variable	Overall
Age (years)	35.37 ± 4.51
Body weight (kg)	66.78 ± 14.29
Height (cm)	164.09 ± 6.75
BMI (kg/m^2^)	24.74 ± 5.29
AMH (ng/mL)	3.48 ± 3.15
FSH (IU)	6.76 ± 2.90
AFC	21.17 ± 14.36
Duration of infertility (years)	2.21 ± 2.13
Estradiol day 6 (ng/mL)	1651.07 ± 1847.22
Estradiol ovulation trigger (ng/mL)	8943.72 ± 5655.81
Progesterone trigger or −1 day (ng/mL)	3.30 ± 1.54
Stimulation day trigger (days)	11.93 ± 1.68
Follicles > 14 mm last US	11.78 ± 6.47
Oocytes retrieved	15.47 ± 9.53

Data are presented as a mean ± standard deviation or percentage of the total. AFC, antral follicle count; AMH, anti-müllerian hormone; BMI, body mass index; FSH, follicle stimulating hormone; IU, international unit.

Baseline male patient characteristics are presented in Table 2. On average, men were two years older than women. Over 50% reported alcohol consumption. In most cases (68.83%), autologous sperm was collected through ejaculation. In the remaining cases, the sperm was surgically aspirated (19.35%) or donated (11.82%).

**Table 2:** Demographics and baseline reproductive characteristics of male participants.

Variable	Overall
Age (years)	37.88 ± 7.22
Sperm concentration (M)	38.80 ± 53.40
Sperm motility a + b (%)	36.88 ± 24.42
Sperm morphology (%)	7.38 ± 14.89
DNA fragmentation (%)	11.41 ± 14.54
Tabaco	19.29%
Alcohol	64.69%
Alcohol	64.69%

Data are presented as a mean ± standard deviation or percentage of the total.

The biochemical pregnancy, clinical pregnancy, and live birth rates were 51.17%, 42.52%, and 35.74%, respectively. Patients delivered infants weighing 3061.19 ± 982.66 g at 37.29 ± 2.7 weeks gestation.

### 3.2. AI Model Shows High Discriminating Power for Classifying Transferrable Embryos

**Figure 3:**
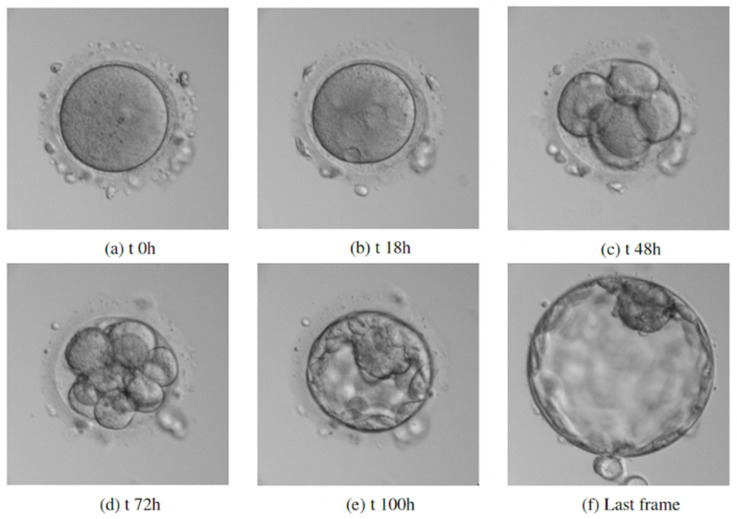
Key developmental stages of human embryos.

We first evaluated the ability of our AI model to classify embryos as transferrable or not based on a single ovum pick-up. Analyzing 2330 embryo images from 259 patients, the model achieved 94.2% accuracy using a single image. When incorporating all six developmental keyframes (Figure 3), the model reached a 97.9% concordance with the ground truth, which was defined as the embryologist’s decision. This underscores the model’s strong discriminating power in identifying embryos suitable for transfer.

### 3.3. AI Model Accurately Predicts Pregnancy Based on Embryo Time-Lapse Images and Clinical Data

Table 3 presents different metrics for the predictions of live birth, clinical pregnancy, and biochemical pregnancy when the model input involved either 35 clinical variables or embryo images (with only one frame and six frames) alone, or the combination of clinical and embryo data.

**Table 3:** Pregnancy AI model performance metrics.

Clinical Data					
	Accuracy	AUC	Precision	Sensitivity	F1-score
Live birth	67.26%	0.65	64.1%	66.7%	63.8%
Clinical Pregnancy	68.42%	0.61	67.1%	60.7%	64.5%
Biochemical pregnancy	67.67%	0.63	64.1%	66.7%	63.8%
Single frame accuracy					
	Accuracy	AUC	Precision	Sensitivity	F1-score
Live birth	54.23%	0.56	61.1%	60.9%	59.8%
Clinical Pregnancy	58.64%	0.63	63.7%	60.7%	58.5%
Biochemical pregnancy	65.65%	0.60	64.2%	66.7%	61.8%
Multiple frames accuracy					
	Accuracy	AUC	Precision	Sensitivity	F1-score
Live birth	57.45%	0.67	71.5%	68.1%	69.3%
Clinical Pregnancy	67.34%	0.68	69.5%	67.1%	68.4%
Biochemical pregnancy	72.45%	0.69	75.5%	71.4%	71.5%
Combination frame + clinical data accuracy					
	Accuracy	AUC	Precision	Sensitivity	F1-score
Live birth	71.98%	0.76	77.1%	76.3%	76.2%
Clinical Pregnancy	73.45%	0.77	79.1%	77.1%	77.1%
Biochemical pregnancy	77.87%	0.79	83.1%	79.3%	80.3%

The pregnancy AI model predicted which embryos led to a live birth with a modest 65% accuracy based on the patient’s clinical data alone. The accuracy of predictions for biochemical and clinical pregnancy was not significantly different when considering the clinical parameters alone (67.67% and 68.42%, respectively). In contrast, when only single images of transferrable transferred embryos were used (1.3 embryos were transferred per patient), the model predicted live births with 54.23% accuracy. Single images were useful for predicting biochemical pregnancy (65.65% accuracy), but the model’s performance was slightly lower than when the clinical parameters were used. Interestingly, the six time-lapse frames of embryo development were the best predictors of biochemical pregnancy (72.45% accuracy) but poor predictors of live birth. (57.45% accuracy). The model performed best when combining data from the 35 clinical variables with the six key frames of embryo development, with a remarkable 71.98% accuracy of live birth prediction, showing the power of integrating multiple images with clinical information. The size of the dataset was insufficient to perform further stratification to assess its efficacy on specific patient groups.

### 3.4. Important Biological Features Recognized by the AI Model

**Figure 4:**
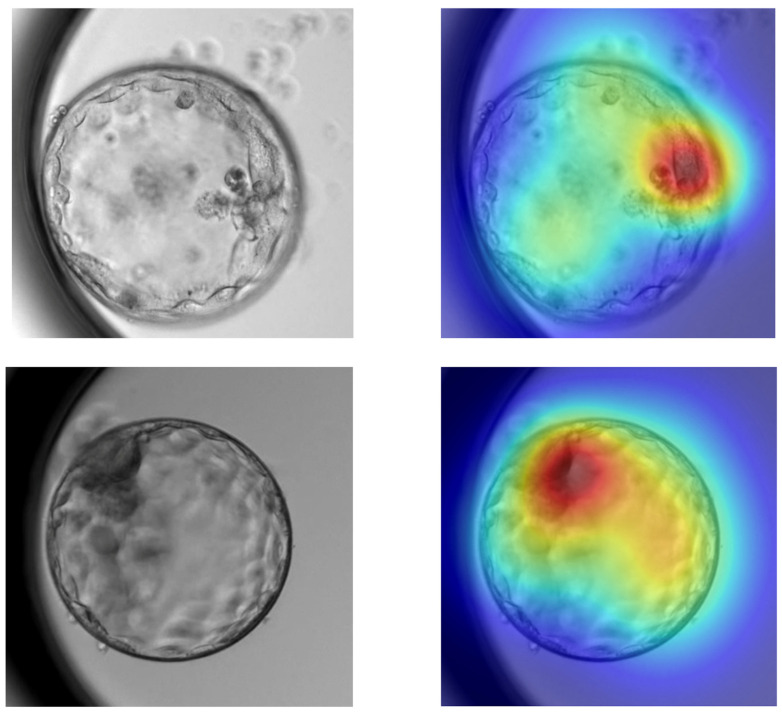
Two representative Grad-CAM visualizations showing regions contributing to the CNN predictions. Warmer colors indicate regions of higher importance, often highlighting the inner cell mass and trophectoderm.

Grad-CAM visualizations showed that the CNN primarily focused on biologically meaningful regions of the blastocyst, including the inner cell mass and trophectoderm while also capturing global morphological features. In some cases, the model’s attention extended to less relevant regions, indicating occasional suboptimal focus (Figure 4).

### 3.5. AI Model Could Boost the Theoretical Probability of Pregnancy on the First Transfer

We used a mathematical equation to determine if our model can decrease the time to pregnancy by boosting the probability of success at the first transfer. In cases where patients have three transferrable blastocysts of the same quality, each associated with a 35.7% probability of live birth (which is the probability derived from the SETs of the internal cohort we analyzed), it could take three transfers to achieve a live birth rate of 73%. Alternatively, with an AI-assisted transfer, the patients would have a 72% probability of live birth at the first transfer, significantly shortening the time to pregnancy. Importantly, these cases are based on the model metrics in ideal situations, and represent hypothetical cases that will not apply with the same efficacy in all patients.

## 4. Discussion

This study evaluates the effectiveness of integrating IVF patients’ clinical data and embryo development time-lapse images to predict live birth outcomes and embryo availability for treatment. We found that parental characteristics and IVF cycle data contribute significantly to the predictive AI model, synergizing with embryo development imaging data to predict transferrable embryos with 97.88% accuracy and live birth with 72% accuracy.

The accuracy of pregnancy predictions computed by our AI model corroborates those of existing models for predicting biochemical and clinical pregnancy [16,17] (global overview in Supplementary Table S2). However, previous models were generally not predicting the live birth rate, which represents a more valuable and complex outcome.

Acquiring and preprocessing unstructured data from video sources is challenging and time-consuming due to variations in the original data acquisition systems. We devised a new and automated protocol to acquire images, which has become the industry standard [18]. Other studies developed useful automations for processing images from time-lapse systems but they mainly extract blastocyst features. Given recent evidence that day 3 embryo grading can help improve the chances of a live birth following day 5 embryo transfer [19,20], our work included representative images of all key stages of embryo development which could not be leveraged with blastocyst-optimized software [21].

Our model, when using only clinical data from patients, did not perform particularly well, for both the prediction of live birth and implantation, as already observed in recent work [22]. These findings point towards a logic underperformance of this type of models, as the clinical variables cannot be used alone, especially if the algorithm is aimed at embryo selection. Analogously, the use of a single frame of the embryo did not provide a great improvement, as the highest accuracy was only a little above 57% in predicting live birth, while it performed already much better in predicting implantation. The use of multiple frames further improved the prediction of a biochemical pregnancy or successful implantation. However, when predicting live birth, the model’s performance only exceeded random guessing (50% chance of an accurate prediction) by 4% with single frames or 7% with multiple frames. This suggests that blastocyst images may be best suited for predicting implantation or establishment of pregnancy.

Finally, the most sensible increase in our predictive chances of live birth was when we combined clinical parameters and embryo images, which substantially increased the accuracy and the AUC, surpassing the algorithms that report live birth rates as outcome (Supplementary Table S2).

This study focused on two main outcomes that are clinically relevant for patients and providers alike. The first one is the prediction of a live birth, which we were able to predict with an accuracy of 71.98%, thus providing an interesting tool to counsel patients and manage expectations at the beginning of treatment.

The second outcome of interest is the prediction of the availability of an embryo for treatment, i.e., its ability to develop at least to the blastocyst stage with a certain level of quality so that it can be used in the clinical course for the patient. Our predictive model performed strongly in mirroring embryologists’ decisions regarding embryo viability, achieving a commendable accuracy of 97.98%. This prediction affords the possibility to reduce, in specific instances, the time to pregnancy, mainly for cases where the presence of multiple embryos of the same quality would force a random choice from the embryologist. However, the time to pregnancy shortening is calculated on our internal cohort, and the situations we reported are selected hypothetical cases based on an internal cohort that might limit the shortening of time and number of transfers needed.

Finally, the findings from this study call for an in-depth evaluation of existing models based exclusively on time-lapse imaging, as reporting clinical pregnancy might have overestimated their predictive value. Nevertheless, the ability to predict live birth rate provides the most clinical utility and should be the main objective of all future prediction models.

The use of such combined algorithms could benefit the clinical practice in multiple ways. AI-assisted embryo ranking systems can enhance objectivity and consistency across embryologists and clinics, particularly in high-volume centers where inter-observer variability may affect outcomes. The availability of a validated model capable of predicting live birth, rather than solely implantation or clinical pregnancy, could improve patient counseling by providing patient specific predictions before transfer. In the long term, incorporating AI-driven predictions into electronic medical records and laboratory management systems could also facilitate real-time decision support, standardize embryo selection protocols, and promote a data-driven approach to reproductive medicine

### Limitations and Future Directions

We recognize some weaknesses of the present study. The major limitations are the lack of external validation in diverse cohorts and the number of transferred embryos we used to train the model. Regarding the former, this work represent the foundation to preoceed with further multicentric validations that will provide an answer on its potential and versatility as tool for clinicians, because at the moment, its internal-only validation, limits its applicability and its potential. For the number of embryos used, due to the relatively reduced n, we could not perform stratification based on the type of fertilization, as the IVF group was relatively small (25%) and it could not give rise to meaningful analysis. In addition, in this subset, there is no useful information on the initial 8 h, and we acknowledge this could somehow limit the prediction capacity. However, we can hypothesize that the initial phases of development will have less weight on the final outcome, and represent a fraction less than 10% of the total time.

We also could not stratify by factors such as age, clinical stimulation, of fresh and frozen transfers as the number of samples could not cover all the different groups and provide robust results.

However, these limitations are partially countered by the cross-sectional nature of our sample, including gametes of different origins (partner vs. donor) and source (testicular and ejaculate), four types of controlled ovarian stimulation protocols, and fertilization techniques (IVF or ICSI), increasing its applicability to a non-selected population. Further, we included only cycles with embryos transferred at the blastocyst stage (day 5 or 6) to enhance dataset consistency and reliability. Due to this, this approach may not translate well for patients with shorter embryo cultures or a significantly worse prognosis. While our study utilized a CNN-based architecture, we acknowledge the rapid advancements in deep learning, particularly the emergence of Vision Transformers (ViTs), which have shown promising results in various medical image prediction tasks. Finally, antoher relevant limitation is the retrospective nature of the dataset used to develop the AI model.

While this allows faster access to large amounts of data and the initial development of a potential clinical module, the findings from this study will have to undergo prospective validation from independent patient populations to increase generalization, and possibly compared or integrated with novel advanced architectures to further enhance predictive capabilities and methodological relevance, and possibly also by increasing the number of clinical parameters to include to find the best set of predictor and optimize its outputs.

## 5. Conclusions

Our study highlights the potential of combining clinical data and embryo images to enhance predictive models in reproductive medicine. Future work should focus on validating this model in multicentric studies to include diverse patient populations and increase its generalizability. Also, new technical integrations together with additional data types could improve prediction accuracy.

## Data Availability

The code used is publicly available at https://github.com/jminano/Pregnancy-AI.

## Supplementary Materials

The following supporting information can be downloaded at https://www.mdpi.com/article/10.3390/medicina62020364/s1, Supplementary Table S1. List of clinical variables used as model predictors. Supplementary Table S2. State of the ART of the most recent AI based model to predict cycle outcomes. References [23,24,25,26,27,28] are cited in the supplementary materials.

## References

[1] Kushnir V.A., Smith G.D., Adashi E.Y. (2022). The Future of IVF: The New Normal in Human Reproduction. Reprod. Sci..

[2] Liu K.E., Case A. (2017). No 346-Advanced Reproductive Age and Fertility. J. Obstet. Gynaecol. Can..

[3] Gardner D.K., Schoolcraft W.B. (1999). Culture and transfer of human blastocysts. Curr. Opin. Obstet. Gynecol..

[4] Rhenman A., Berglund L., Brodin T., Olovsson M., Milton K., Hadziosmanovic N., Holte J. (2015). Which set of embryo variables is most predictive for live birth? A prospective study in 6252 single embryo transfers to construct an embryo score for the ranking and selection of embryos. Hum. Reprod..

[5] Desai N., Goldberg J.M., Austin C., Falcone T. (2018). Are cleavage anomalies, multinucleation, or specific cell cycle kinetics observed with time-lapse imaging predictive of embryo developmental capacity or ploidy?. Fertil. Steril..

[6] Esteva A., Kuprel B., Novoa R.A., Ko J., Swetter S.M., Blau H.M., Thrun S. (2017). Dermatologist-level classification of skin cancer with deep neural networks. Nature.

[7] McKinney S.M., Sieniek M., Godbole V., Godwin J., Antropova N., Ashrafian H., Back T., Chesus M., Corrado G.S., Darzi A. (2020). International evaluation of an AI system for breast cancer screening. Nature.

[8] Kanakasabapathy M.K., Sadasivam M., Singh A., Preston C., Thirumalaraju P., Venkataraman M., Bormann C.L., Draz M.S., Petrozza J.C., Shafiee H. (2017). An automated smartphone-based diagnostic assay for point-of-care semen analysis. Sci. Transl. Med..

[9] Fanton M., Nutting V., Solano F., Maeder-York P., Hariton E., Barash O., Weckstein L., Sakkas D., Copperman A.B., Loewke K. (2022). An interpretable machine learning model for predicting the optimal day of trigger during ovarian stimulation. Fertil. Steril..

[10] Salih M., Austin C., Warty R.R., Tiktin C., Rolnik D.L., Momeni M., Rezatofighi H., Reddy S., Smith V., Vollenhoven B. (2023). Embryo selection through artificial intelligence versus embryologists: A systematic review. Hum. Reprod. Open.

[11] Fruchter-Goldmeier Y., Kantor B., Ben-Meir A., Wainstock T., Erlich I., Levitas E., Shufaro Y., Sapir O., Har-Vardi I. (2023). An artificial intelligence algorithm for automated blastocyst morphometric parameters demonstrates a positive association with implantation potential. Sci. Rep..

[12] Shen L., Zhang Y., Chen W., Yin X. (2022). The Application of Artificial Intelligence in Predicting Embryo Transfer Outcome of Recurrent Implantation Failure. Front. Physiol..

[13] Barnes J., Brendel M., Gao V.R., Rajendran S., Kim J., Li Q., Malmsten J.E., Sierra J.T., Zisimopoulos P., Sigaras A. (2023). A non-invasive artificial intelligence approach for the prediction of human blastocyst ploidy: A retrospective model development and validation study. Lancet Digit. Health.

[14] World Health Organization (2010). WHO Laboratory Manual for the Examination and Processing of Human Semen.

[15] Girshick R. Fast r-cnn. Proceedings of the IEEE International Conference on Computer Vision.

[16] Kim H.M., Ko T., Kang H., Choi S., Park J.H., Chung M.K., Kim M., Kim N.Y., Lee H.J. (2024). Improved prediction of clinical pregnancy using artificial intelligence with enhanced inner cell mass and trophectoderm images. Sci. Rep..

[17] Wen J.Y., Liu C.F., Chung M.T., Tsai Y.C. (2022). Artificial intelligence model to predict pregnancy and multiple pregnancy risk following in vitro fertilization-embryo transfer (IVF-ET). Taiwan. J. Obstet. Gynecol..

[18] Fjeldstad J., Qi W., Mercuri N., Siddique N., Meriano J., Krivoi A., Nayot D. (2024). An artificial intelligence tool predicts blastocyst development from static images of fresh mature oocytes. Reprod. Biomed. Online.

[19] Herbemont C., Sarandi S., Boujenah J., Cedrin-Durnerin I., Sermondade N., Vivot A., Poncelet C., Grynberg M., Sifer C. (2017). Should we consider day-2 and day-3 embryo morphology before day-5 transfer when blastocysts reach a similar good quality?. Reprod. Biomed. Online.

[20] Xiao Y., Zhang P., Wang L., Ko Y., Wang M., Xi J., Zhou C., Chen X. (2024). Optimizing single blastocyst selection: The role of day 3 embryo morphology in vitrified-warmed blastocyst transfer cycles. Reprod. Biomed. Online.

[21] Chéles D.S., Ferreira A.S., de Jesus I.S., Fernandez E.I., Pinheiro G.M., Dal Molin E.A., Alves W., de Souza R.C.M., Bori L., Meseguer M. (2022). An image processing protocol to extract variables predictive of human embryo fitness for assisted reproduction. Appl. Sci..

[22] Liu X., Chen Z., Ji Y. (2023). Construction of the machine learning-based live birth prediction models for the first in vitro fertilization pregnant women. BMC Pregnancy Childbirth.

[23] Salih M., Austin C., Mantravadi K., Seow E., Jitanantawittaya S., Reddy S., Vollenhoven B., Rezatofighi H., Horta F. (2025). Deep learning classification integrating embryo images with associated clinical information from ART cycles. Sci. Rep..

[24] Borna M.-R., Sepehri M.M., Maleki B. (2024). An artificial intelligence algorithm to select most viable embryos considering current process in IVF labs. Front. Artif. Intell..

[25] Sun L., Li J., Zeng S., Luo Q., Miao H., Liang Y., Cheng L., Sun Z., Tai W.H., Han Y. (2024). Artificial intelligence system for outcome evaluations of human in vitro fertilization-derived embryos. Chin. Med. J..

[26] Mina A., Younesi M., Doohandeh T., Darzi S., Ardehjani N.A., Sheibani S., Hosseinirad H., Valizadeh R. (2025). Predicting pregnancy outcomes in IVF cycles: A systematic review and diagnostic meta-analysis of artificial intelligence in embryo assessment. Contracept. Reprod. Med..

[27] Khosravi P., Kazemi E., Zhan Q., Malmsten J.E., Toschi M., Zisimopoulos P., Sigaras A., Lavery S., Cooper L.A.D., Hickman C. (2019). Deep learning enables robust assessment and selection of human blastocysts after in vitro fertilization. npj Digit. Med..

[28] Peng J., Geng X., Zhao Y., Hou Z., Tian X., Liu X., Xiao Y., Liu Y. (2024). Machine learning algorithms in constructing prediction models for assisted reproductive technology (ART) related live birth outcomes. Sci. Rep..

