# Supplementary material for: Pregnancy AI: Development and Internal Validation of an Artificial Intelligence Tool to Predict Live Births in ICSI and IVF Cycles Using Clinical Features and Embryo Images
Source: Medicina (Kaunas). 2026 Feb 12;62(2):364. doi: 10.3390/medicina62020364 (PMC12943628; doi:10.3390/medicina62020364)
Supplement: Supplementary file 1 [file medicina-62-00364-s001.zip › medicina-4076013-supplementary.pdf]

Supplementary Table S1. List of clinical variables used as model predictors

Variable	Description	Type
Maternal_Age	Years at time of IVF cycle	Continuous
Maternal_BMI	Body mass index (kg/m²)	Continuous
AMH	Anti-Müllerian hormone level (ng/mL)	Continuous
Antral_Follicle_Count	Number of antral follicles on ultrasound	Continuous
Maternal_Smoking	Smoking status (yes/no)	Categorical
Maternal_Alcohol	Alcohol consumption (yes/no)	Categorical
Maternal_Caffeine	Caffeine consumption (yes/no)	Categorical
Maternal_Ethnicity	Ethnic background (e.g., Caucasian, Asian)	Categorical
Maternal_Infertility_Duration	Years of infertility	Continuous
Maternal_Primary_Diagnosis	Primary infertility cause (e.g., ovulatory, tubal)	Categorical
Maternal_Prior_Pregnancies	Number of prior pregnancies	Continuous
Maternal_Prior_Live_Births	Number of prior live births	Continuous
Maternal_FSH	Follicle-stimulating hormone level (IU/L)	Continuous
Maternal_Estradiol_Baseline	Baseline estradiol level (pmol/L)	Continuous
Paternal_Age	Years at time of IVF cycle	Continuous
Paternal_BMI	Body mass index (kg/m²)	Continuous
Paternal_Smoking	Smoking status (yes/no)	Categorical
Paternal_Alcohol	Alcohol consumption (yes/no)	Categorical
Paternal_Sperm_Source	Sperm origin (ejaculated/surgical/donor)	Categorical
Paternal_Sperm_Concentration	Sperm concentration (million/mL)	Continuous
Paternal_Sperm_Motility	Percentage of motile sperm	Continuous
Paternal_Sperm_Morphology	Percentage of normal morphology	Continuous
Cycle_Type	IVF or ICSI	Categorical
Stimulation_Protocol	GnRH antagonist/agonist/SMART	Categorical
Gonadotropin_Dose	Total gonadotropin dose (IU)	Continuous
Stimulation_Duration	Days of stimulation	Continuous
Estradiol_Trigger	Estradiol level at trigger (pmol/L)	Continuous
Progesterone_Trigger	Progesterone level at trigger (ng/mL)	Continuous
Oocyte_Count	Number of oocytes retrieved	Continuous
Mature_Oocyte_Count	Number of mature (MII) oocytes	Continuous
Fertilization_Rate	Percentage of oocytes fertilized	Continuous
Embryo_Count	Number of embryos formed	Continuous
Blastocyst_Count	Number of blastocysts formed	Continuous
Transfer_Type	Fresh or frozen transfer	Categorical
Endometrial_Thickness	Endometrial thickness at transfer (mm)	Continuous

Supplementary Table S2. State of the ART of the most recent AI based model to predict cycle outcomes

	Input data	Cohort size & setting	Outcome(s)	AI architecture	Performance metrics	Comments
**Salih et al., 2025**	19 clinical variables + blastocyst images	1,503 cycles from 6 IVF centres	Clinical pregnancy	MLP for clinical data, CNN for images, fusion model	MLP: accuracy 81.76 %, AUC 0.91; CNN: accuracy 66.89 %, AUC 0.73	Demonstrated benefit of integrating clinical and image data.
**Kim et al., 2024**	Static day-5 blastocyst images (ICM and TE enhanced)	2,555 blastocysts from 7 IVF centres	Clinical pregnancy	CNN with region enhancement and attention mapping	Enhanced model AUC 0.741 vs baseline 0.716; model focused on ICM and TE regions in 99 % of cases	Showed interpretability via attention maps. Did not include clinical variables.
**Borna et al., 2024**	3 static images per embryo	252 time lapse videos	Embryo quality (development to blastocyst)	CNN with segmentation and transfer learning	Accuracy 75 % (multiple images); Dice coefficient 93.21 % for segmentation	Demonstrated that multiple images and segmentation improve performance. No pregnancy outcomes reported.
**Sun et al., 2024**	Maternal clinical features + day-3/day-5 embryo metrics	193 cycles; prospective evaluation	Implantation (clinical pregnancy)	Ensemble AI model (likely random forest + CNN)	Day-5 model: **AUC 0.769**, accuracy higher than embryologists (55 % vs 40.7 %)	Combined maternal and embryo features outperformed human assessment. No external validation reported.
**Life Whisperer & FiTTE systems (Mina et**	Static blastocyst images ± clinical data	Life Whisperer: 21,600 images; FiTTE:	Clinical pregnancy	CNN and deep residual networks with optional	Life Whisperer accuracy 64.3 %; FiTTE accuracy 65.2 %, AUC 0.70	Commercial tools with modest accuracy; not openly available.

	Input data	Cohort size & setting	Outcome(s)	AI architecture	Performance metrics	Comments
**al., 2025 review)**		19,342 images		clinical features		
**Koshravi et al., 2019**	Time-lapse images of morula/blastocyst development	>50,000 embryos across multiple clinics	Embryo grading, euploidy, fetal heartbeat	CNN and attention networks	Accuracy up to 97.5 % for extreme grades; AUC 0.66–0.70 for ICM and TE predictions	High accuracy for stratified grading; moderate AUCs for detailed outcomes.
**Peng et al., 2024**	19 clinical variables (maternal age, BMI, hormone levels)	11,938 couples from China	Live birth	Random forest, logistic regression	AUROC 0.671 (random forest), 0.674 (logistic regression)	Demonstrates that clinical data alone have moderate predictive power.

References

Salih M, Austin C, Warty RR, et al. Deep learning classification integrating embryo images with associated clinical information from ART cycles. *Scientific Reports*. 2025;15(1):2076. doi:10.1038/s41598-025-02076-x.

Kim HM, Ko T, Kang H, et al. Improved prediction of clinical pregnancy using artificial intelligence with enhanced inner cell mass and trophectoderm images. *Scientific Reports*. 2024;14(1):3240. doi:10.1038/s41598-024-52241-x.

Borna, M.-R., Sepehri, M. M., & Maleki, B. (2024). An artificial intelligence algorithm to select most viable embryos considering current process in IVF labs. Frontiers in Artificial Intelligence, 7, 1375474. https://doi.org/10.3389/frai.2024.1375474

Sun L, Li J, Zeng S, et al. Artificial intelligence system for outcome evaluations of human in vitro fertilization-derived embryos. *Chinese Medical Journal*. 2024;137(16):1939-1949. doi:10.1097/CM9.0000000000003162.

Mina A, Younesi M, Doohandeh T, et al. Predicting pregnancy outcomes in IVF cycles: a systematic review and diagnostic meta-analysis of artificial intelligence in embryo assessment. *Contraception and Reproductive Medicine*. 2025;10(1):59. doi:10.1186/s40834-025-00400-4.

Khosravi P, Kazemi E, Zhan Q, et al. Deep learning enables robust assessment and selection of human blastocysts after in vitro fertilization. *npj Digital Medicine*. 2019;2(1):21. doi:10.1038/s41746-019-0096-y.

Peng, J., Geng, X., Zhao, Y., et al. (2024). Machine learning algorithms in constructing prediction models for assisted reproductive technology (ART) related live birth outcomes. Scientific Reports, 14, 32083. https://doi.org/10.1038/s41598-024-83781-x
